# *Helminthostachys zeylanica* Water Extract Ameliorates Airway Hyperresponsiveness and Eosinophil Infiltration by Reducing Oxidative Stress and Th2 Cytokine Production in a Mouse Asthma Model

**DOI:** 10.1155/2020/1702935

**Published:** 2020-12-02

**Authors:** Wen-Chung Huang, Nai-Chun Ting, Yu-Ling Huang, Li-Chen Chen, Chwan-Fwu Lin, Chian-Jiun Liou

**Affiliations:** ^1^Graduate Institute of Health Industry Technology, Research Center for Food and Cosmetic Safety, and Research Center for Chinese Herbal Medicine, College of Human Ecology, Chang Gung University of Science and Technology, Taoyuan City 33303, Taiwan; ^2^Division of Allergy, Asthma, and Rheumatology, Department of Pediatrics, Chang Gung Memorial Hospital, Linkou, Guishan Dist., Taoyuan City 33303, Taiwan; ^3^Graduate Institute of Clinical Medical Sciences, Chang Gung University, Taoyuan City 33303, Taiwan; ^4^National Research Institute of Chinese Medicine, Ministry of Health and Welfare, Beitou, Taipei, Taiwan; ^5^Department of Pediatrics, New Taipei Municipal Tu Cheng Hospital, Chang Gung Memorial Hospital and Chang Gung University, Taiwan; ^6^Department of Cosmetic Science, Research Center for Food and Cosmetic Safety, and Research Center for Chinese Herbal Medicine, College of Human Ecology, Chang Gung University of Science and Technology, Taoyuan City 33303, Taiwan; ^7^Department of Anesthesiology, Chang Gung Memorial Hospital, Linkou, Guishan Dist., Taoyuan City 33303, Taiwan; ^8^Department of Nursing, Division of Basic Medical Sciences, Research Center for Chinese Herbal Medicine, Chang Gung University of Science and Technology, Taoyuan City 33303, Taiwan

## Abstract

*Helminthostachys zeylanica* is a traditional folk herb used to improve inflammation and fever in Taiwan. Previous studies showed that *H. zeylanica* extract could ameliorate lipopolysaccharide-induced acute lung injury in mice. The aim of this study was to investigate whether *H. zeylanica* water (HZW) and ethyl acetate (HZE) extracts suppressed eosinophil infiltration and airway hyperresponsiveness (AHR) in asthmatic mice, and decreased the inflammatory response and oxidative stress in tracheal epithelial cells. Human tracheal epithelial cells (BEAS-2B cells) were pretreated with various doses of HZW or HZE (1 *μ*g/ml–10 *μ*g/ml), and cell inflammatory responses were induced with IL-4/TNF-*α*. In addition, female BALB/c mice sensitized with ovalbumin (OVA), to induce asthma, were orally administered with HZW or HZE. The result demonstrated that HZW significantly inhibited the levels of proinflammatory cytokines, chemokines, and reactive oxygen species in activated BEAS-2B cells. HZW also decreased ICAM-1 expression and blocked monocytic cells from adhering to inflammatory BEAS-2B cells *in vitro*. Surprisingly, HZW was more effective than HZE in suppressing the inflammatory response in BEAS-2B cells. Our results demonstrated that HZW significantly decreased AHR and eosinophil infiltration, and reduced goblet cell hyperplasia in the lungs of asthmatic mice. HZW also inhibited oxidative stress and reduced the levels of Th2 cytokines in bronchoalveolar lavage fluid. Our findings suggest that HZW attenuated the pathological changes and inflammatory response of asthma by suppressing Th2 cytokine production in OVA-sensitized asthmatic mice.

## 1. Introduction

Allergic asthma is a complex airway inflammatory disease, where antigens stimulate and induce airway hyperresponsiveness (AHR), activated leukocyte infiltration, and airway mucosal hyperplasia [1, 2]. During acute attacks of asthma, patients suddenly coughed, wheezed, and experienced chest tightness, shortness of breath, and dyspnea [3]. When an allergen is inhaled into airways, it stimulates the epithelial cells to release inflammatory cytokines and mediators for respiratory inflammation [3]. In addition, airway epithelial cells secrete chemokines to attract more inflammatory immune cells and infiltrate into lung tissues, causing a more severe inflammatory response [4]. Furthermore, inflamed epithelial cells are accompanied by oxidative damage to epithelial cells and lung tissue, as well as increased activation of immune cells to destruct lung function [5, 6]. In particular, infiltrate of pulmonary macrophages and neutrophils can induce oxygen-dependent cytotoxic effects to release more O_2_^−^ against the invasion of allergens [7]. Moreover, the oxidizing substance released by those immune cells also causes pulmonary oxidative damage [8]. Therefore, reducing the inflammation and oxidative damage of airways would improve symptoms during the development of asthma in patients.

Recent studies found that the allergen-stimulated Th2 cell activity of the lungs could be an important mechanism in the development of chronic asthma [9]. Th2 cells could secrete IL-4, IL-5, and IL-13 to trigger asthma symptom, including increased eosinophil infiltration and AHR [4, 10]. Therefore, blocking the activity of Th2 cells may ameliorate asthma in patients.

Current clinical strategies include treatment with asthma medications that contain acute attack medication and prevention medication [11]. Smooth muscle vasodilators that reduce the airway contraction and allow patients to breathe can be effective in severe asthma. Moreover, patients may use oral corticosteroids for maintenance and prevention of asthma attacks [12]. The dosage of oral corticosteroids is slight with few serious side effects [13]. Steroids are immunosuppressants and inhibit the function of Th1 and Th2 cells [14]. Therefore, the general public is not treated with steroid drugs for asthma.

Traditional Chinese medicine, yoga, qigong, and homeopathy are very common alternative medicines [15]. Chinese medicine has widely been used for thousands of years, and there have been many herbal formulas, including Ding Chuan Tang and Xiao-Qing-Long-Tang, used to improve asthma symptoms in China and Taiwan [16–18]. These herbal formulas contained ephedra, which contains ephedrine that predominantly reduced smooth muscle contraction in asthmatic patients [19]. Yet, ephedrine is a refined amphetamine and may endanger the health of teenagers [20]. Hence, scholars and practitioners have studied and dispensed alternative herbal formulas to replace those traditional formulas. Many scholars found that other herbal formulas could attenuate AHR and eosinophil infiltration by suppressing the activity of Th2 cells in asthmatic mice, including MSSM-002 and Danggui Buxue Tang [21, 22].

*Helminthostachys zeylanica* (L.) Hook. (Ophioglossaceae) is a common anti-inflammatory folk herb in Taiwan and has been used to improve inflammatory diseases and as an antipyretic [23]. Ugonin K was isolated from *H. zeylanica* and had anti-inflammatory effects in lipopolysaccharide- (LPS-) stimulated macrophages [24]. *H. zeylanica* extracts could also decrease MMP-9 expression and the inflammatory response in bradykinin-induced brain astrocytes [25]. We also found that water extract of *H. zeylanica* could reduce LPS-induced acute lung injury [26]. However, it is not clear whether *H. zeylanica* could attenuate the inflammatory response of airways and improve asthma symptoms. In this study, we investigated whether the *H. zeylanica* ethyl acetate (HZE) extract and water (HZW) extract could reduce AHR and eosinophil infiltration in asthmatic mice, and decrease oxidative stress and inflammatory response in tracheal epithelial cells.

## 2. Materials and Methods

### 2.1. *H. zeylanica* Extraction

The roots of H. zeylanica were collected from Taipei, Taiwan, in July 2013. The plant was identified by comparison with the voucher specimen (NRICM-99-003) already deposited at the herbarium of National Research Institute of Chinese Medicine, Taiwan. The roots of *H. zeylanica* (1000 g) were heated with ethanol at 50°C for 4 h. After evaporation of the solvent, the ethanol extract was partitioned between water and ethyl acetate to give water extract (HZW: 23.8 g, yield 2.38%) and ethyl acetate extract (HZE: 14.2 g, yield 1.42%).

### 2.2. HPLC Analysis of HZW

HZW (7.1 mg) was dissolved in 2 ml methanol and responded by ultrasonic wave for 10 minutes at 25°C. After the filtration through a 0.45 *μ*m polyvinylidene fluoride membrane, the filtrate was injected into the Agilent 1100 series HPLC (10 *μ*l) system. The separation was performed on a COSMOSIL 5C_18_-AR-II column (5 *μ*m, 250 × 4.6 mm, i.d.), which was eluted at a flow rate of 1.0 ml/min with a mobile phase gradient of *A*–*B* (*A* = H_2_O (0.05% formic acid), *B* = MeOH) varying as follows: 0–10 min: 90–80% *A*, 10–20% *B*; 10–40 min: 80–0% *A*, 20–100% *B*; and 40–50 min: 0–0% *A*, 100–100% *B*. The UV detection wavelength was set at 254 nm. The two standards are quercetin-3-*O*-*β*-D-glucopyranosyl-4′-*O*-*β*-D-glucopyranosyl-(1 → 2)-*β*-D-glucopyranoside (compound 1) and quercetin-4′-*O*-*β*-D-glucopyranosyl-(1 → 2)-*β*-D-glucopyranoside (compound 2) [27].

### 2.3. HZW and HZE Treatment of BEAS-2B Cells

HZW was dissolved in PBS and HZE was dissolved in DMSO. The concentration of these stock solutions was 10 mg/ml. There was ≤0.1% DMSO in the experimental culture medium. BEAS-2B cells (American Type Culture Collection, Manassas, VA, USA) were seeded in DMEM/F12 medium in cultured plates. The cells were pretreated with HZW or HZE (1-10 *μ*g/ml) for 1 h, and then treated with 10 ng/ml TNF-*α* and 10 ng/ml IL-4 for 24 h. The supernatants were collected, and cytokines or chemokines were detected using specific ELISA kits. Cell viability was measured using the MTT assay, and the results were determined by the absorbance values at 570 nm using a microplate reader (Multiskan FC, Thermo, Waltham, MA, USA).

### 2.4. Detection of Reactive Oxygen Species (ROS) Production

BEAS-2B cells were stimulated with TNF-*α*/IL-4 and treated with HZW or HZE in 96-well plates for 24 h. Cells were then treated with 2′,7′-dichlorofluorescin diacetate (DCFH-DA) (Catalog No. D6883; Sigma, St. Louis, MO, USA) for 30 min as described previously [28, 29]. Cells were then lysed for detection of ROS using a multimode microplate reader (BioTek Synergy HT, Bedfordshire, United Kingdom).

### 2.5. Cell-Cell Adhesion Assay

BEAS-2B cells were treated with various doses of HZW or HZE for 1 h and then stimulated with TNF-*α*/IL-4 for 24 h in 6-well plates as described previously [30, 31]. THP-1 cells (Bioresource Collection and Research Center, Taiwan) were treated with calcein-AM solution (Catalog No. C1359; Sigma) at 37°C for 30 min, and THP-1 cells were cocultured with BEAS-2B cells at 37°C for 30 min. Finally, THP-1 cells adhered to BEAS-2B cells were measured using fluorescence microscopy (Olympus, Tokyo, Japan).

### 2.6. Experimental Animals

The animals used in this study were 6- to 8-week-old female BALB/c mice, obtained from the National Laboratory Animal Center in Taiwan. Water and food were provided ad libitum, and mice were housed at constant temperature and humidity environment, and maintained on a 12 h light/dark cycle at the Animal Center of Chang Gung University, Taiwan. Animal experimental procedures were approved by the Laboratory Animal Care Committee of Chang Gung University of Science and Technology (IACUC approval number: 2013-001).

### 2.7. Allergy Sensitization and Drug Treatment

Asthma was induced in mice by 50 *μ*g ovalbumin (OVA) (Catalog No. A5503; Sigma) and 0.8 mg aluminum hydroxide (Thermo, Rockford, IL, USA) in 200 *μ*l normal saline via intraperitoneal injection on days 1-3 and 14, as described previously [32]. Mice were challenged and exposed with aerosolized 2% OVA using an ultrasonic nebulizer (DeVilbiss Pulmo-Aide 5650D, USA) for 20 min on days 14, 17, 20, 23, and 27. From day 14 to day 27, the mice were orally administered with saline (N and OVA groups), HZW, or HZE for 14 consecutive days. In every independent experiment, mice were divided into six groups (of 10 mice each): (1) normal control mice (N group) sensitized with normal saline; (2) OVA-sensitized control mice (OVA group) sensitized with OVA; (3-5) experimental group mice sensitized with OVA and orally administered with 1 mg/kg, 5 mg/kg, or 10 mg/kg HZW or HZE (named HZW1, HZW5, and HZW10 or HZE1, HZE5, and HZE10, respectively); and (6) prednisolone control (P group) OVA-sensitized mice orally administered with 5 mg/kg prednisolone.

### 2.8. Measurement of AHR

On day 28, the AHR of mice was measured to evaluate airway function via various inhaled doses of methacholine, as described previously [33]. All mice inhaled aerosolized methacholine (0-40 mg/ml) for 3 min, respectively. A single-chamber, whole-body plethysmograph (Buxco Electronics, Troy, NY, USA) was used to record the values of enhanced pause (Penh), a measurement of AHR.

### 2.9. Serum Collection and Splenocyte Cultures

On day 29, mice were anesthetized with 4% isoflurane (Aesica, Queenborough, UK), and blood was collected from the orbital vascular plexus and centrifuged at 6000 rpm for 5 min at 4°C. The serum was collected to measure OVA-specific antibodies that were detected by ELISA as previously described [28]. Next, mice were sacrificed by CO_2_ asphyxiation. Their splenocytes were isolated and cultured with 100 *μ*g/ml OVA in RPMI 1640 medium for 5 continuous days. The levels of cytokines in the supernatants were assayed as previously described [32].

### 2.10. Bronchoalveolar Lavage Fluid (BALF) and Cell Collection

Mice were anesthetized using 4% isoflurane, and BALF was collected as described previously [34]. Briefly, the mice were incubated with an indwelling needle into the trachea, and the lungs were washed three times using 1 ml normal saline. The supernatant was collected to detect the levels of cytokines and chemokines by ELISA. Additionally, Giemsa stain (Catalog No. GS500; Sigma) was used to stain cells for measuring cell counts and differentiated cell morphology.

### 2.11. Histologic Analysis of Lung Tissue

Lung tissues were embedded in paraffin and cut into 6 *μ*m thick sections. Hematoxylin and eosin (HE) staining was used to observe the eosinophil infiltration of the lungs. Eosinophil infiltration inflammatory index used fivepoint scoring system as described previously [35]. Briefly, the degree of cell infiltration was scored as follows: 0, no cell; 1, a few cells; 2, a ring of inflammatory cells of one cell layer; 3, a ring of inflammatory cells of two to four cell layers; and 4, a ring of inflammatory cells > four cell layers. Furthermore, periodic acid-Schiff (PAS) staining was used to evaluate goblet cell hyperplasia of the trachea as described previously [28]. The lung tissue slide was deparaffinized and stained with periodic acid solution for 5 min. Next, the slide was washed and added with Schiff's reagent for 15 min. Finally, hematoxylin solution was added, and the goblet cells were observed using a light microscope (Olympus, Tokyo, Japan).

### 2.12. Glutathione (GSH) Assay

A glutathione assay kit was used to determine the glutathione levels of the lungs, according to the manufacturer's instructions (Catalog No. CS0260; Sigma). 50 mg lung tissues was homogenized by homogenizer (FastPrep-24, MP Biomedicals, Santa Ana, CA, United States). The sample was centrifuged, and supernatant detected the level of total glutathione, including reduced GSH and glutathione disulfide using a multimode microplate reader (BioTek Synergy HT, Bedfordshire, United Kingdom).

### 2.13. Malondialdehyde (MDA) Assay

The lipid peroxidation assay kit (Catalog No. MAK085; Sigma) was used to evaluate the MDA level of the lungs, according to manufacturer's instructions. Lung tissues were homogenized by homogenizer (FastPrep-24, MP Biomedicals, Santa Ana, CA, United States). The supernatant was treated with perchloric acid for protein precipitation. The tissue solution was centrifuged and collected supernatant to detect MDA expression by a multimode microplate reader (BioTek Synergy HT).

### 2.14. Lung RNA Isolation and Real-Time PCR

Lung tissues were homogenized and RNA extracted with TRIzol reagent (Catalog No. 15596026; Life Technologies, Carlsbad, CA, USA), as described previously [34, 36]. We used RNA to synthesize cDNA and a spectrofluorometric thermal cycler (iCycler; Bio-Rad) to investigate specific gene expression. The reaction cycling conditions were as follows: preincubated samples at 95°C for 10 min. Next, the PCR was performed as 40 cycles of 95°C for 15 seconds and 60°C for 1 minute.

### 2.15. ELISA

Cytokines, chemokines, and ICAM-1 were detected in the BALF and supernatants of cell cultures using specific ELISA kits, according to the manufacturer's instructions (R&D Systems, Minneapolis, MN, USA), as previously described [33]. Serum OVA-IgE and OVA-IgG1 were measured with specific ELISA kits (BD Biosciences). The OVA-IgG1 standard curve was used to calculate the units of IgG1, and the levels of OVA-IgE were determined by OD 450.

### 2.16. Statistical Analysis

Statistical analyses were performed with SPSS v19 (SPSS, Chicago, IL, USA). Animal experiment results were analyzed by one-way analysis of variance (ANOVA), followed by the Dunnett and post hoc test for multiple comparisons. Between two groups, an unpaired Student *t*-test was used in cell experiment. Statistical significance was set at *P* < 0.05, and the results were expressed as the mean ± standard error of the mean (SEM) from three independent experiments.

## 3. Results

### 3.1. HZW Inhibited the Levels of Cytokines and Chemokines in Inflammatory BEAS-2B Cells

**Figure 1:**
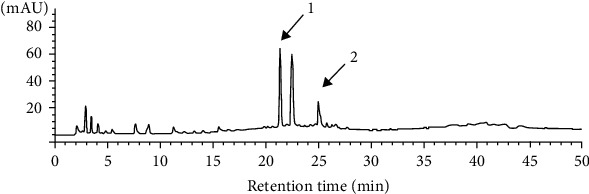
HPLC chromatograms of *H. zeylanica* water (HZW) extract. 1: quercetin-3-*O-β*-D-glucopyranosyl-4′-*O*-*β*-D-glucopyranosyl-(1 → 2)-*β*-D-glucopyranoside; 2: quercetin-4′-*O*-*β*-D-glucopyranosyl-(1 → 2)-*β*-D-glucopyranoside.

**Figure 2:**
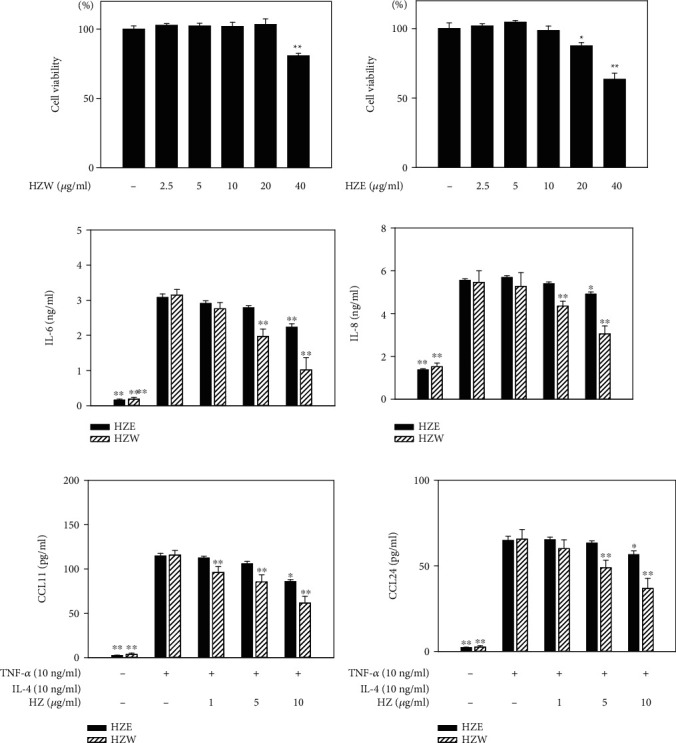
The effects of HZW and HZE on cytokine and chemokine productions in BEAS-2B cells. Cell viability of (a) HZW and (b) HZE in BEAS-2B cells. The levels of IL-6 (c), IL-8 (d), CCL11 (e), and CCL24 (f) were measured by ELISA. All data are presented as the mean ± SEM. ^∗^*P* < 0.05 and ^∗∗^*P* < 0.01 compared with BEAS-2B cells stimulated with TNF-*α*/IL-4. HZ: *Helminthostachys zeylanica*. HZW was dissolved in PBS and HZE was dissolved in DMSO. Hence, vehicle HZE experiment contained 0.1% DMSO in medium and vehicle HZW experiment did not contain DMSO in medium.

The HPLC fingerprint of *H. zeylanica* water (HZW) extract (Figure 1) showed compound 1 and compound 2 elute at 21.2 and 25.2 minutes, which was specific enough to be used for the identification of *H. zeylanica* [27]. The cytotoxicity of HZW and HZE in BEAS-2B cells was determined by MTT assay. Cell viability was not significantly affected by the doses of HZW or HZE ≤ 10 *μ*g/ml (Figures 2(a) and 2(b)). Therefore, in this current experiment, we used HZW and HZE doses from 1 *μ*g/ml to 10 *μ*g/ml *in vitro*. We used TNF-*α*/IL-4 to stimulate inflammation in BEAS-2B cells to evaluate the anti-inflammatory effect of HZW and HZE. Our experimental results demonstrated that HZW could significantly reduce the levels of IL-6 and IL-8 at 5 and 10 *μ*g/ml (Figures 2(c) and 2(d)). We also found that HZW significantly reduced eotaxin (CCL11 and CCL24) production at 5 and 10 *μ*g/ml (Figures 2(e) and 2(f)). However, HZE was not as effective at reducing eotaxins and proinflammatory cytokines, although 10 *μ*g/ml HZE could significantly inhibit IL-6, IL-8, and CCL11 production. We also found that the 10 *μ*g/ml HZW group significantly decreased the levels of IL-6, IL-8, CCL11, and CCL24 compared with the 10 *μ*g/ml HZE group. Thus, HZW was better in reducing the inflammatory response than HZE.

### 3.2. HZW Regulated ROS Production in BEAS-2B Cells

**Figure 3:**
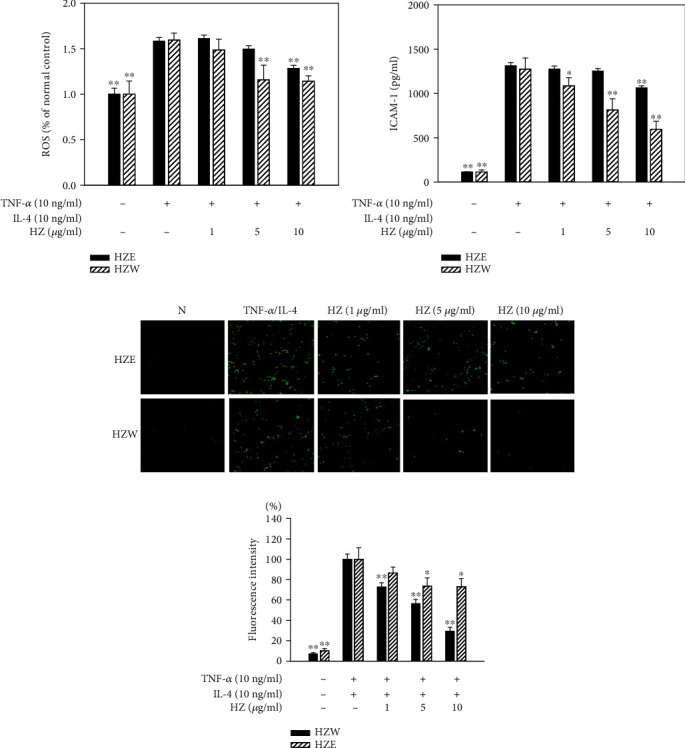
HZW and HZE inhibited ROS and THP-1 cell adherence to the activated BEAS-2B cells. (a) Percentage of ROS detected in cells treated with HZW or HZE compared with TNF-*α*/IL-4-activated BEAS-2B. (b) The levels of ICAM-1, detected by ELISA, in BEAS-2B cells activated with TNF-*α*/IL-4. (c) Fluorescence microscopy images of THP-1 cells labeled with calcein-AM and mixed with normal (N) and TNF-*α*/IL-4-activated BEAS-2B cells, in the absence or presence of HZW or HZE. HZ: *Helminthostachys zeylanica*. (d) Fluorescence intensity of THP-1 cell adhesion to BEAS-2B cells. The data are presented as the mean ± SEM. ^∗^*P* < 0.05 and ^∗∗^*P* < 0.01 compared with BEAS-2B cells stimulated with TNF-*α*/IL-4.

BEAS-2B cells were treated with HZW or HZE and then stimulated with TNF-*α*/IL-4. Cells were lysed and detected with ROS using a multimode microplate reader. We found HZW decreased the ROS levels more than HZE in TNF-*α*/IL-4-activated BEAS-2B cells (Figure 3(a)).

### 3.3. HZW Reduced Monocytic Cell Adhesion to BEAS-2B Cells

HZW significantly suppressed the levels of intercellular adhesion molecule 1 (ICAM-1) compared with HZE in TNF-*α*/IL-4-activated BEAS-2B cells (Figure 3(b)). Additionally, HZW reduced the THP-1 cells that adhered to inflammatory BEAS-2B cells (Figures 3(c) and 3(d)), while HZE did not.

### 3.4. HZW Improved AHR and Reduced the Inflammatory Cells in the BALF of OVA-Sensitized Mice

**Figure 4:**
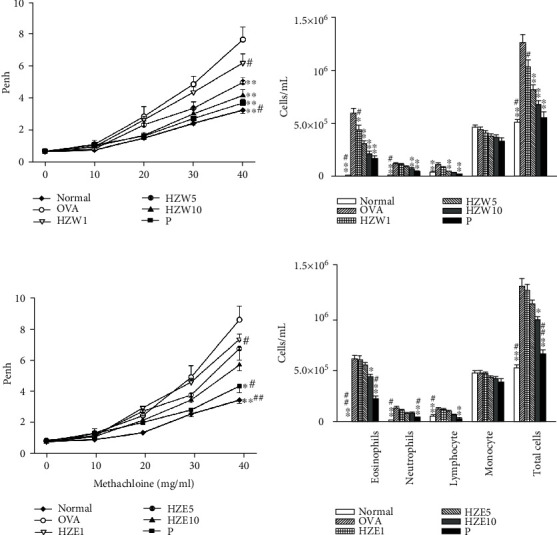
Effect of HZW or HZE on AHR and cell counts in the BALF of asthmatic mice. The AHR (Penh values) was measured after inhalation of increasing methacholine doses (10-40 mg/ml) in normal (N) and OVA-stimulated (OVA) mice, treated with or without HZW (a) or HZE (c) (*n* = 10 mice/group, measured in three independent experiments). The numbers of inflammatory cells and total cells in the BALF from OVA-sensitive mice, treated with or without HZW (b) or HZE (d). All data are presented as the mean ± SEM. ^∗^*P* < 0.05and ^∗∗^*P* < 0.01 compared with the OVA control group. ^#^*P* < 0.05 and ^##^*P* < 0.01 compared to the HZW10 or HZE10 group. Prednisolone (P) control orally administered with 5 mg/kg prednisolone as positive control.

AHR is one of the important features of asthma. It mainly evaluates the respiratory function when allergen or stimulus induces to increase sensitivity and reactivity of airways [37]. The AHR assay result showed that OVA-sensitized mice have Penh values that are increased in a dose-dependent manner compared with the normal mice. At 40 mg/ml methacholine, the HZW groups had significantly reduced the value of Penh compared with the OVA group (Figure 4(a)) (HZW1: 6.22 ± 0.56, *P* = 0.08; HZW5: 5.01 ± 0.30, *P* < 0.01; HZW10: 4.18 ± 0.38, *P* < 0.01 vs. OVA: 7.68 ± 0.39). In HZE-treated OVA-sensitized mice, only HZE10 could suppress Penh values compared with the OVA group (Figure 4(c)) (HZE1: 7.33 ± 0.35, *P* = 0.75; HZE5: 6.74 ± 0.73, *P* = 0.31; HZE10: 5.68 ± 0.38, *P* < 0.05 vs. OVA: 8.58 ± 0.44). Prednisolone-treated OVA-sensitized mice also significantly inhibited the Penh values compared to the OVA group mice. We also investigated whether HZW and HZE could suppress inflammatory cells in the BALF of asthmatic mice. The result showed that HZW5 and HZW10 could significantly inhibit eosinophils and total cells compared with the OVA group (Figure 4(b)). However, HZE did not significantly inhibit eosinophils and total cells compared with the OVA group (Figure 4(d)). Prednisolone-treated OVA-sensitized mice also significantly inhibited eosinophils and total cells compared to the OVA-sensitized mice. Hence, we only analyzed how HZW ameliorates the effects of inflammation, oxidation, and the immune mechanism of asthma in the animal model.

### 3.5. HZW Regulated the MDA and GSH Levels in the Lungs

**Figure 5:**
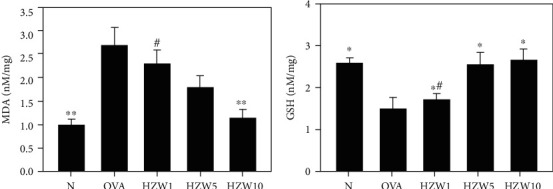
The effects of HZW on oxidative stress factors. The MDA level (a) and GSH activity (b) were measured in the lung tissues of mice. Data are presented as the mean ± SEM. ^∗^*P* < 0.05 and ^∗∗^*P* < 0.01 compared with the OVA control group. ^#^*P* < 0.05 compared to the HZW10 group.

Asthma attacks would induce oxidative stress for causing lung cell damage [38]. We found that OVA-sensitized mice had significantly enhanced the MDA levels and reduced GSH production in lung tissue compared with normal mice (Figures 5(a) and 5(b)). HZW suppressed MDA and increased the GSH levels compared with OVA mice.

### 3.6. HZW Attenuated Eosinophil Infiltration and Goblet Cell Hyperplasia in the Lungs

**Figure 6:**
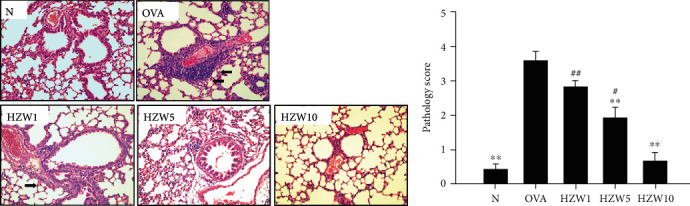
The effect of HZW on eosinophil infiltration of lung tissue in OVA-sensitized mice. (a) Hematoxylin and eosin staining of lung sections (6 *μ*m) observed eosinophil infiltration (200x magnification). Eosinophils were indicated with arrows. (b) The scoring of inflammation *via* pathological evaluation in lung sections. Data are presented as the mean ± SEM. ^∗^*P* < 0.05 and ^∗∗^*P* < 0.01 compared with the OVA control group. ^#^*P* < 0.05 and ^##^*P* < 0.01 compared to the HZW10 group.

**Figure 7:**
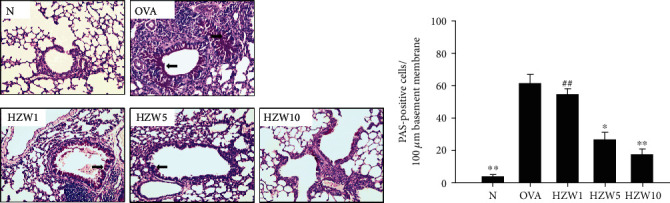
The effects of HZW on goblet cell hyperplasia in the lung tissue of OVA-sensitized mice. (a) Lung sections (6 *μ*m) were stained with PAS to observe goblet cell hyperplasia (200x magnification). Goblet cells were indicated with arrows. (b) Results expressed as the number of PAS-positive cells per 100 *μ*m of the basement membrane. Data are presented as the mean ± SEM. ^∗^*P* < 0.05 and ^∗∗^*P* < 0.01 compared with the OVA control group. ^##^*P* < 0.01 compared to the HZW10 group.

In the lungs, infiltration of inflammatory cells is an important factor to cause pulmonary inflammation [39]. There were more eosinophils infiltrating between the blood vessels and bronchus of the lungs in OVA-sensitive mice than normal mice, and HZW could suppress eosinophil infiltration of the lungs in asthmatic mice (Figure 6). The PAS staining also showed HZW could decrease goblet cell hyperplasia of airways compared with OVA-sensitive mice (Figure 7).

### 3.7. HZW Regulated Cytokine and Chemokine Expression in BALF and Lung Tissue

**Figure 8:**
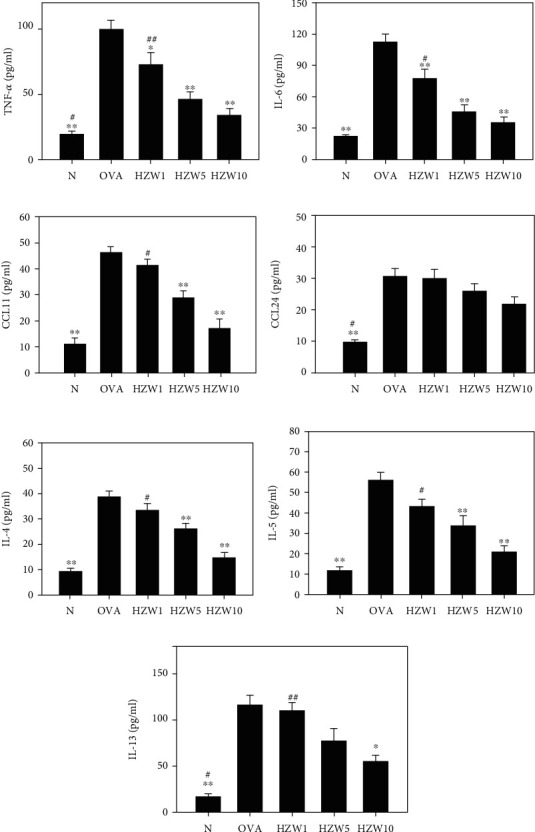
The effects of HZW on the levels of cytokines and chemokines in the BALF. The concentrations of TNF-*α* (a), IL-6 (b), CCL11 (c), CCL24 (d), IL-4 (e), IL-5 (f), and IL-13 (g) were measured by ELISA. All data are presented as the mean ± SEM. ^∗^*P* < 0.05 and ^∗∗^*P* < 0.01 compared with the OVA control group. ^#^*P* < 0.05 and ^##^*P* < 0.01 compared to the HZW10 group.

**Figure 9:**
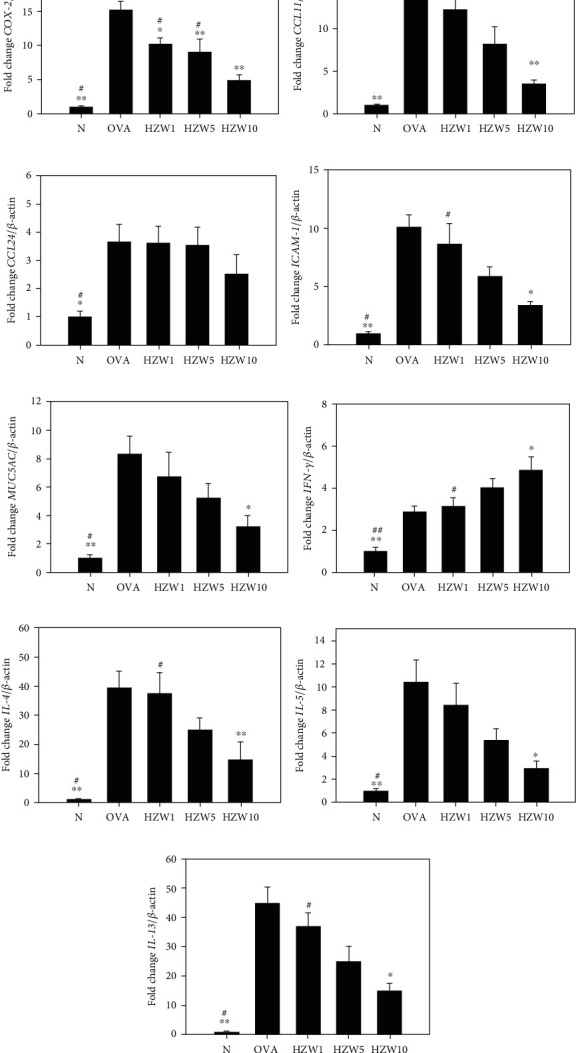
Effects of HZW on gene expression of cytokines, chemokines, and inflammatory mediators in the lungs. The gene expression levels of COX-2 (a), CCL11 (b), CCL24 (c), ICAM-1 (d), MUC5AC (e), IFN-*γ* (f), IL-4 (g), IL-5 (h), and IL-13 (i) were determined by real-time PCR of RNA extracted from the lung tissues of normal (N) and OVA-stimulated (OVA) mice, treated with or without HZW. Fold changes in expression were measured relative to *β*-actin expression (internal control). Data are presented as the mean ± SEM. ^∗^*P* < 0.05 and ^∗∗^*P* < 0.01 compared with the OVA control group. ^#^*P* < 0.05 and ^##^*P* < 0.01 compared to the HZW10 group.

To analyze the levels of proinflammatory cytokine and Th2-associated cytokines in BALF, cytokines and chemokines were detected using ELISA. We found that HZW-treated mice had significantly suppressed the TNF-*α* levels compared with OVA-sensitized mice (Figure 8). HZW also significantly reduced IL-6, CCL11, IL-4, IL-5, and IL-13 compared with the OVA group, but did not decrease the levels of CCL24 in the BALF of asthmatic mice. The gene expression in lung tissues measured by real-time PCR demonstrated that HZW significantly reduced COX-2, CCL11 (not CCL24), ICAM-1, MUC5AC, IL-4, IL-5, and IL-13 gene expression compared with OVA-sensitized mice (Figure 9). However, HZW also increased IFN-*γ* gene expression in asthmatic mice.

### 3.8. HZW Regulated the Antibody and Cytokine Levels in Serum and Splenocytes

**Figure 10:**
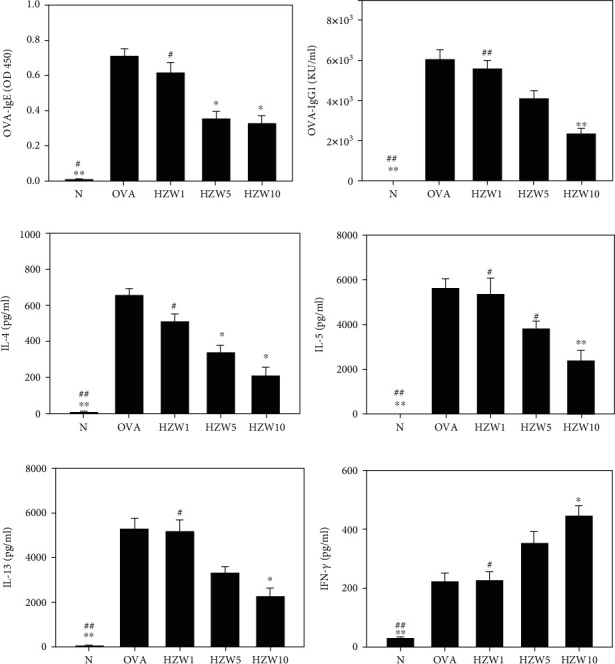
Effect of HZW on the levels of the OVA-specific antibodies and cytokines. HZW reduced the levels of OVA-IgE (a) and OVA-IgG1 (b) in serum as well as IL-4 (c), IL-5 (d), IL-13 (e), and IFN-*γ* (f) produced by OVA-activated splenocytes. All data are presented as the mean ± SEM. ^∗^*P* < 0.05 and ^∗∗^*P* < 0.01 compared with the OVA control group. ^#^*P* < 0.05 and ^##^*P* < 0.01 compared to the HZW10 group.

To analyze the levels of OVA-specific antibodies in serum, HZW significantly attenuated the OVA-IgG1 and OVA-IgE levels compared with OVA-sensitized mice (Figures 10(a) and 10(b)). We also analyzed the levels of Th2-associated cytokines in splenocyte culture supernatants and found that mice treated with HZW10 had significantly reduced the levels of IL-4, IL-5, and IL-13 (Figures 10(c)–10(e)). Moreover, HZW increased the IFN-*γ* levels compared with OVA-sensitized mice (Figure 10(f)).

## 4. Discussion

Previous studies confirmed that *H. zeylanica* could improve liver damage in Wistar rats [40]. Previously, we found that HZW could also decrease inflammatory responses in lung tissues from LPS-treated mice [26]. In this current study, we found that HZW is more effective than HZE to reduce inflammatory response and decrease the ROS levels in TNF-*α*/IL-4-activated BEAS-2B cells. Also, HZW inhibited THP-1 cell adherence to inflammatory BEAS-2B cells more than HZE. We thought that HZW fraction should contain the main substance or compound that inhibited the inflammatory response of human tracheal epithelial cells. In the present study, we found that HZW significantly reduced oxidative stress and suppressed Th2-associated cytokines and chemokines in BALF. HZW could also inhibit the gene expression of inflammatory chemokines and cytokines in lung tissues. In addition, HZW significantly attenuated tracheal goblet cell hyperplasia, eosinophil infiltration of the lungs, and ameliorated AHR in asthmatic mice. Furthermore, HZW decreased the levels of Th2 cytokines in splenocyte culture medium and OVA-specific IgE in serum.

Recently, a study demonstrated that ugonin J and ugonin K were the main flavonoids of HZE [26]. A previous study found that HZW analyzed with HPLC, and the UV detection wavelength was set at 254 nm. HZW could be isolated two bioactivity compounds, including quercetin-4-*O-β*-D-glucopyranosyl-(1 → 2)-*β*-D-glucopyranoside and quercetin-3-*O-β*-D-glucopyranosyl-4-*O-β*-D-glucopyranosyl-(1 → 2)-*β*-D-glucopyranoside [27]. These two pure compounds could suppress production of nitric oxide and proinflammatory cytokines in LPS-stimulated macrophage [27]. We thought that these two compounds should also have the potential to ameliorate respiratory inflammation and AHR and in asthmatic mice.

Asthma is a respiratory inflammatory disease. When patients inhale allergens, they stimulate an inflammatory response of airway epithelial cells [3]. Those epithelial cells then secrete inflammatory cytokines and chemokines for invasions of foreign substances [6]. Our experimental findings that HZW is effective at reducing the IL-6 levels in inflammatory tracheal epithelial cells suggest it may help prevent the development of lung inflammation in asthmatic patients. Asthmatic mice, treated with HZW, had suppressed the levels of IL-6 and TNF-*α* in BALF, and also reduced gene expression of inflammatory mediators (COX-2) in the lungs. Hence, HZW is a good natural plant extract with an anti-inflammatory effect in asthmatic mice.

During the asthma development process, immune cells and epithelial cells induce oxidative stress leading to lung tissue damage [41]. Researchers have found that patients could regulate oxidative stress, reduce allergic inflammation, and suppress asthma by taking an antioxidant supplement [42]. A previous study found that oral administration of high dose vitamin C could reduce inflammation and oxidative stress of the lungs and eosinophilic infiltration of BALF in OVA-sensitized mice [43]. Licochalcone A and quercetin also were found that could improve asthma by suppressing ROS and inflammation in OVA-induced asthmatic mice [44, 45]. Therefore, antioxidants have great potential to improve asthma symptoms. The antioxidant enzyme GSH can provide oxidation protection by reducing oxidative damage and the chronic inflammatory response in the lungs of asthmatic mice [38]. Lipid peroxidation influences cellular function via oxidative damage and eventually leads to cell death [38]. Our results showed that HZW could effectively reduce ROS in tracheal epithelial cells and decrease the levels of MDA, a lipid peroxidation marker, in the lung tissue in asthmatic mice. Furthermore, HZW increased GSH expression to increase the protective effects against oxidative damage. Hence, HZW was effective at reducing lung damage by suppressing oxidative stress in asthmatic mice.

Excessive Th2 cytokines exacerbate the pathology response of asthma by increasing AHR, goblet cell hyperplasia, excessive mucus secretion, and inflammatory cell infiltration [4, 46]. Previous studies also found that IL-13 could aggravate AHR in asthmatic mice, causing shortness of breath and bronchoconstriction [1]. In clinical asthma cases, there are high IL-13 levels observed in BALF that induce AHR and difficulty of breathing [47]. Another study found that IL-13 knockout mice did not have significantly increased AHR compared with wild-type asthmatic mice [48]. We found that HZW could significantly decrease IL-13 gene expression in the lungs and the IL-13 levels in BALF, and improve AHR in asthmatic mice. Furthermore, Th2 cells secrete excess IL-4 to induce B cell activation and secretion of more IgE to bind to the IgE*ε*RI receptor of mast cells [3]. When patients inhale allergens, they form a complex with IgE and mast cells to induce mast cell activation and release histamine and leukotrienes, causing severe lung inflammation and allergic responses [6]. We found that HZW could significantly decrease the IL-4 levels and suppress OVA-specific IgE production in serum. These results confirm that HZW was effective at reducing the acute inflammatory response of airways and decreasing allergic response in asthmatic mice.

When combined with IL-13, IL-4 can promote goblet cell hyperplasia of the airway, and those goblet cells secrete excessive mucus leading to airway obstruction, breathing difficulties, and even death in asthmatic patients [18]. A previous study found that IL-4 or IL-13 knockout mice did not significantly increase goblet cell hyperplasia of the airway compared with wild-type asthmatic mice [9]. Here, we demonstrated that HZW reduced IL-4 and IL-13 production and decreased goblet cell hyperplasia in lung tissue, and also suppressed Muc5Ac gene expression in the lungs for improved mucus secretion.

Recently, a study demonstrated that active Th2 cells release more IL-5 to induce eosinophil proliferation and differentiation from the bone marrow [39]. In asthmatic patients, airway epithelial cells also secrete more eotaxins (CCL11, CCL24, and CCL26) to attract eosinophils that migrate into the lung tissue [6]. Eosinophils release more inflammatory substances, including eosinophil cationic protein and eosinophil peroxidase, to cause lung inflammation and oxidation damage in lung tissue [24]. HZW could significantly suppress CCL11 and IL-5 expression in the lungs and BALF, and also decrease the levels of CCL11 and CCL24 in inflammatory tracheal epithelial cells for reduced eosinophil differentiation and attraction to lung tissue. Hence, we thought that HZW mainly reduced CCL11 secretion of the lungs to decrease eosinophil infiltration in the lungs of asthmatic mice. In addition, HZW reduced ICAM-1 expression of tracheal epithelial cells that adhere to eosinophils and migrate into lung tissue to reduce the inflammation and allergic response in the lungs. In the current study, we confirmed that HZW inhibited the activity of Th2 cells, reducing lung inflammation and allergic reactions in asthmatic mice.

Steroids are clinical drugs for treating or preventing asthma [12]. However, steroids interfered with the function of immune cells and inhibited the expression of Th1 and Th2 cells [14]. Interferon-*γ*, a Th1 cytokine, would suppress expression in asthmatic patient. In the present study, the gene expression of lung tissues demonstrated that HZW could increase IFN-*γ* gene expression in asthmatic mice. Clinical trials found that herbal formulas, Ding Chuan Tang and ASHMI, reduced asthma symptoms and improved respiratory function by regulating the activity of Th1/Th2 cells [17, 49]. In this study, HZW inhibited allergic reactions and improved lung inflammation in asthmatic mice. Interestingly, HZW did not suppress the activity of Th2 and Th1 cells as steroids. In steroid-resistant asthmatic patients, tracheal epithelial cells would release IL-8, thereby attracting more neutrophils into the lungs to cause serious inflammatory reactions. Our study found that the levels of IL-8 attenuated after BEAS-2B cells treated with HZW, indicating that HZW might reduce the inflammatory response in asthmatic patients.

In conclusion, our results demonstrated that HZW, but not HZE, significantly suppressed AHR, mucus hypersecretion, and eosinophil infiltration via blocked Th2 cytokine production in asthmatic mice. HZW also reduced oxidative stress and inflammation to prevent lung damage and maintain the function of the respiratory system. Thus, HZW may potentially ameliorate inflammation and antioxidative stress in asthma.

## Data Availability

The data used to support the findings of this study are included with the article.

## Abbreviations

AHR: Airway hyperresponsiveness
BALF: Bronchoalveolar lavage fluid
DCFH-DA: 2′,7′-Dichlorofluorescin diacetate
GSH: Glutathione
HE: Hematoxylin and eosin staining
HZW: *Helminthostachys zeylanica* water
HZE: *Helminthostachys zeylanica* ethyl acetate extracts
LPS: Lipopolysaccharide
OVA: Ovalbumin
MDA: Malondialdehyde
PAS: Periodic acid-Schiff
Penh: Enhanced pause
ROS: Reactive oxygen species.
